# Second-victim experience in anesthesia and intensive care settings: Organizational gaps, support preferences, and recommendations for action – a cross-sectional study

**DOI:** 10.1097/MD.0000000000043375

**Published:** 2025-07-25

**Authors:** Pinar Ayvat, Ali Galip Ayvat, Gunal Bilek, Ozlem Oner, Gulsah Sehitoglu Alpagut, Omer Faruk Sonmez

**Affiliations:** a Anesthesiology and Reanimation Department, Faculty of Medicine, Izmir Democracy University, Izmir, Turkey; b Project Management Department, Izmir Project Agency, Izmir, Turkey; c Business Management Department, Faculty of Economics and Administrative Sciences, Izmir Democracy University, Izmir, Turkey; d Department of Anesthesiology and Reanimation, Izmir Buca Seyfi Demirsoy Education and Training Hospital, Izmir, Turkey; e Department of Emergency Medicine, Izmir Buca Seyfi Demirsoy Education and Training Hospital, Izmir, Turkey; f Division of Population Health, School of Medicine and Population Health, University of Sheffield, Sheffield, UK.

**Keywords:** anesthesia, intensive care, second-victim, SVEST

## Abstract

Healthcare professionals involved in the management of adverse events often experience psychological and physical distress, known as the “second victim experience.” Anesthesia and intensive care settings are particularly vulnerable to such occurrences due to the high-stakes, and highly stressful work being performed by healthcare professionals. This study aimed to assess the prevalence and dimensions of second-victim experiences among anesthesia and intensive care professionals in Türkiye, while also identifying support preferences and organizational deficiencies. A cross-sectional study was conducted with 175 healthcare professionals working in anesthesia and intensive care units in Türkiye. The Turkish adaptation of the second-victim experience and support tool (T-SVEST) was used to assess psychological distress, physical distress, professional self-efficacy, perceived support mechanisms, and outcome variables such as turnover intentions and absenteeism. Psychometric analyses, confirmatory factor analysis, and correlation/regression tests were performed to evaluate the scale’s validity and to explore correlations among key variables. The T-SVEST demonstrated strong internal consistency (Cronbach α = 0.86) and an improved factor structure following the removal of low-performing items. High levels of psychological and physical distress significantly associated with professional self-efficacy, absenteeism, and turnover intentions were reported. Supervisor and institutional support were perceived as insufficient, particularly among less experienced staff. Age and work experience were positively correlated with colleague support, while married participants reported significantly lower physical distress. Peer support and professional counseling emerged as the most desired support mechanisms. Second-victim experiences are common and consequential among anesthesia and intensive care professionals in Türkiye. The findings have highlighted substantial gaps in organizational support structures and a strong demand for evidence-based peer support systems. Institutional efforts must focus on building a culture of safety and compassion through structured interventions, supervisor training, and proactive debriefing practices to mitigate the impact of adverse events on healthcare providers.

## 1. Introduction

Healthcare professionals frequently work in high-stakes environments where adverse patient events occur inevitably. While patient safety initiatives have traditionally focused on preventing harm to patients – the “first victims” – there is growing recognition that healthcare providers involved in such incidents can also experience significant psychological and emotional distress. These individuals, known as “second victims,” often suffer from symptoms akin to post-traumatic stress disorder, including anxiety, guilt, insomnia, and loss of confidence in their professional abilities.^[1,2]^ The term “second victim” was first introduced by Wu,^[3]^ highlighting that medical errors and adverse patient events impact not only patients but also the well-being and professional performance of healthcare providers.

The second-victim experience and support tool (SVEST), originally developed by Burlison,^[4]^ was designed to assess the experiences of second victims and evaluate the effectiveness of available support mechanisms. The SVEST consists of multiple dimensions, including psychological distress, physical distress, colleague support, supervisor support, institutional support, and professional self-efficacy, along with 2 outcome variables: turnover intentions and absenteeism.^[4]^ Since its initial validation, several cross-cultural adaptations have been developed and validated to reflect different healthcare contexts, including its versions in Argentina, Malaysia, Denmark, Korea, and Türkiye,^[2,5–9]^ among others.

Despite increasing awareness raised by the studies on the second-victim phenomenon, relevant studies focusing on anesthesia and intensive care professionals remain limited. These settings present unique challenges, as healthcare providers frequently make high-risk decisions under intense time constraints, increasing their likelihood of encountering adverse events. In anesthesia and intensive care units, the potential for patient harm is significant, and the emotional burden on providers can be profound. Research suggests that inadequate organizational support systems contribute to increased burnout, professional dissatisfaction, and higher attrition rates among healthcare professionals in these fields.^[1,10]^

To address this gap, this study employed the Turkish adaptation of the SVEST (T-SVEST)^[6,9]^ to assess the second-victim experience among 175 anesthesia and intensive care professionals in Türkiye. The primary aim of this study was to evaluate the prevalence, severity, and dimensions of the second-victim experience in this specific population. Using the validated T-SVEST, we analyzed key aspects such as psychological distress, physical distress, and the perceived adequacy of support mechanisms (e.g., colleague support, supervisor support, institutional support, and professional self-efficacy). Additionally, we examined the impact of second-victim experiences on turnover intentions and absenteeism, which are critical indicators of professional well-being and workforce stability.

A secondary objective was to identify demographic and occupational factors associated with higher second-victim distress. We explored whether age, gender, marital status, educational background, occupation, years of work experience, and workplace type influenced the severity of second-victim experiences. Moreover, this study aimed to determine which support mechanisms were mostly desired by anesthesia and intensive care professionals, providing insights into potential interventions for improving well-being and retention of healthcare professionals.

By systematically assessing these factors, this study seeks to contribute to the development of structured, evidence-based support programs tailored for healthcare professionals working in high-risk environments like anesthesia and intensive care units. The findings will help inform hospital administrators, policymakers, and patient safety advocates about the necessity of comprehensive second-victim support strategies to improve both well-being of healthcare providers and overall patient safety.

## 2. Materials and methods

### 2.1. Study design

This cross-sectional study was conducted in Izmir, Türkiye, in 2024 and included 175 healthcare professionals who were working in anesthesia and intensive care settings. Eligibility criteria for healthcare professionals (physicians, nurses, technicians, or paramedics) were being actively employed in an anesthesia or ICU, having at least 6 months of experience in the current clinical role, and voluntary agreement to participate in the study. Healthcare professionals working in other units (e.g., surgical wards, emergency departments) and those not directly involved in clinical care (e.g., administrative personnel) were not included in the study. Participants were selected from major hospitals (public and university-affiliated) in the region using purposive sampling to ensure participation of a representative sample of professionals from different types of health care institutions. Participation was voluntary and anonymous, with informed consent obtained prior to data collection. Participants were assessed based on gender, age, marital status, educational background, occupation, work experience, and workplace type. Ethical approval was obtained from the noninvasive Clinical Research Ethics Committee of Izmir Democracy University (Approval No: 2023/11-25, Date: December 27, 2023).

Participation was voluntary, anonymous, and independent of workplace authority figures, minimizing social desirability and authority bias. The use of a previously validated and culturally adapted tool (T-SVEST) helped mitigate measurement bias. Participants were recruited from multiple public and university hospitals to enhance representativeness and reduce selection bias.

The sample size was determined based on methodological recommendations for confirmatory factor analysis, which suggest a minimum of 5 to 10 participants per item for structural equation modeling. Given that the SVEST consists of 29 items, a minimum of 145 participants was considered adequate. To ensure sufficient statistical power and account for potential exclusions, we targeted a sample of at least 170 participants. Ultimately, 175 eligible healthcare professionals were included in the final analysis.

### 2.2. SVEST

The SVEST employs a 5-point Likert scale, where higher scores indicate a greater prevalence of second-victim responses and inadequate support resources. The T-SVEST demonstrated strong internal consistency across both validation studies. Koca et al^[9]^ confirmed higher reliability of the T-SVEST across different dimensions with a Cronbach α of 0.90, and subscale values ranging between 0.83 and 0.89. Another validation study,^[6]^ reported a Cronbach α of 0.85, indicating strong overall consistency. These findings confirm that the T-SVEST is a psychometrically sound instrument for assessing second-victim experiences and support mechanisms used in healthcare settings in Türkiye.

### 2.3. Data analysis

All statistical analyses were performed using R. Descriptive statistics, including means, standard deviations, and frequencies, were computed to summarize the characteristics of the participants. In addition, Pearson correlation analysis was conducted to assess correlations between key variables, and results were visualized using correlation heatmaps. Additional statistical tests, including *t*-tests, 1-way ANOVA, and Tukey HSD tests, were applied to examine the impact of demographic and socioeconomic factors on study dimensions. The prospectively determined *P*-value was taken as .05 to display a significant difference (if any) among variables.

The original SVEST scale was developed and validated with a predefined factor structure, which has been confirmed in multiple studies across different cultural contexts. Since the scale was designed with specific dimensions such as psychological distress, supervisor support, and so on, there was no need to explore a new structure using exploratory factor analysis. Instead, confirmatory factor analysis was used to test whether the existing factor structure holds in the current dataset. The datasets generated and/or analyzed during the current study are available from the corresponding author on reasonable request.

## 3. Results

Table 1 shows the characteristics of the participants. The study included 175 participants with a mean age of 42.21 years (SD = 9.62). The majority of the participants were female (55.4%) and married (75.4%). Most participants held a Master’s, PhD, or MD degree (61.7%) and were employed as specialist doctors (41.1%), nurses (27.4%), or academic doctors (16.6%). Work experience varied, with 35.4% having 11 to 20 years and 30.9% having 21 to 30 years. Participants were primarily working in public (46.9%) and university hospitals (41.1%).

**Table 1:** Characteristics of the participants.

Variable	Frequency (n)/percentage (%)
Gender	
Female	97 (55.4%)
Male	78 (44.6%)
Marital status	
Married	132 (75.4%)
Single	43 (24.6%)
Age	Mean = 42.21, SD = 9.62
Educational status	
Associate degree	9 (5.1%)
Bachelor’s degree	58 (33.1%)
Master/PhD/MD	108 (61.7%)
Occupation	
Academic doctor	29 (16.6%)
General practitioner	11 (6.3%)
Nurse	48 (27.4%)
Specialist doctor	72 (41.1%)
Technician/paramedic	15 (8.6%)
Work experience (yr)	
0–10	38 (21.7%)
11–20	62 (35.4%)
21–30	54 (30.9%)
30+	21 (12.0%)
Workplace	
Private	11 (6.3%)
Public health institutions	10 (5.7%)
Public hospital	82 (46.9%)
Public university hospital	72 (41.1%)

To assess the suitability of the dataset for factor analysis, both the Kaiser–Meyer–Olkin measure of sampling adequacy and Bartlett Test of Sphericity were conducted. The overall Kaiser–Meyer–Olkin value was 0.82, which falls within the “good” range, indicating that the dataset is well-suited for factor analysis. The individual MSA (Measure of Sampling Adequacy) values for most items were above 0.70, further confirming the appropriateness of the data. However, a few items exhibited lower MSA values, such as Q11 (0.57), Q12 (0.68), Q17 (0.67), and Q21 (0.67), suggesting that these items may have weaker correlations with the underlying factor structure. Afterwards, Bartlett Test of Sphericity was performed to determine whether the correlation matrix significantly differs from an identity matrix. The test yielded a chi-square (χ²) value of 3692.67 with 630 degrees of freedom and a *P*-value <.001, rejecting the null hypothesis that the correlation matrix is an identity matrix which also confirms that there are significant correlations among variables, further supporting the appropriateness of factor analysis. Given these findings, the dataset meets the assumptions for factor analysis, though the low MSA values for certain items suggest the need for a more refined factor structure.

The reliability analysis of the scale, assessed using Cronbach α, indicates a high level of internal consistency (α = 0.8623), suggesting that the items effectively measure the intended construct. The standardized Cronbach α (0.8593) and Guttman Lambda 6 (0.9376) further confirm the reliability of the scale. However, the average inter-item correlation (0.1451) is relatively low, indicating that some items may not be strongly related to each other, which could affect internal consistency. Despite this, the signal-to-noise ratio (6.1092) and the low standard error of α (0.0142) reinforce the robustness of the scale. Based on these results, while the scale demonstrates strong reliability, a closer examination of individual items may be necessary to enhance internal coherence.

**Figure 1.**
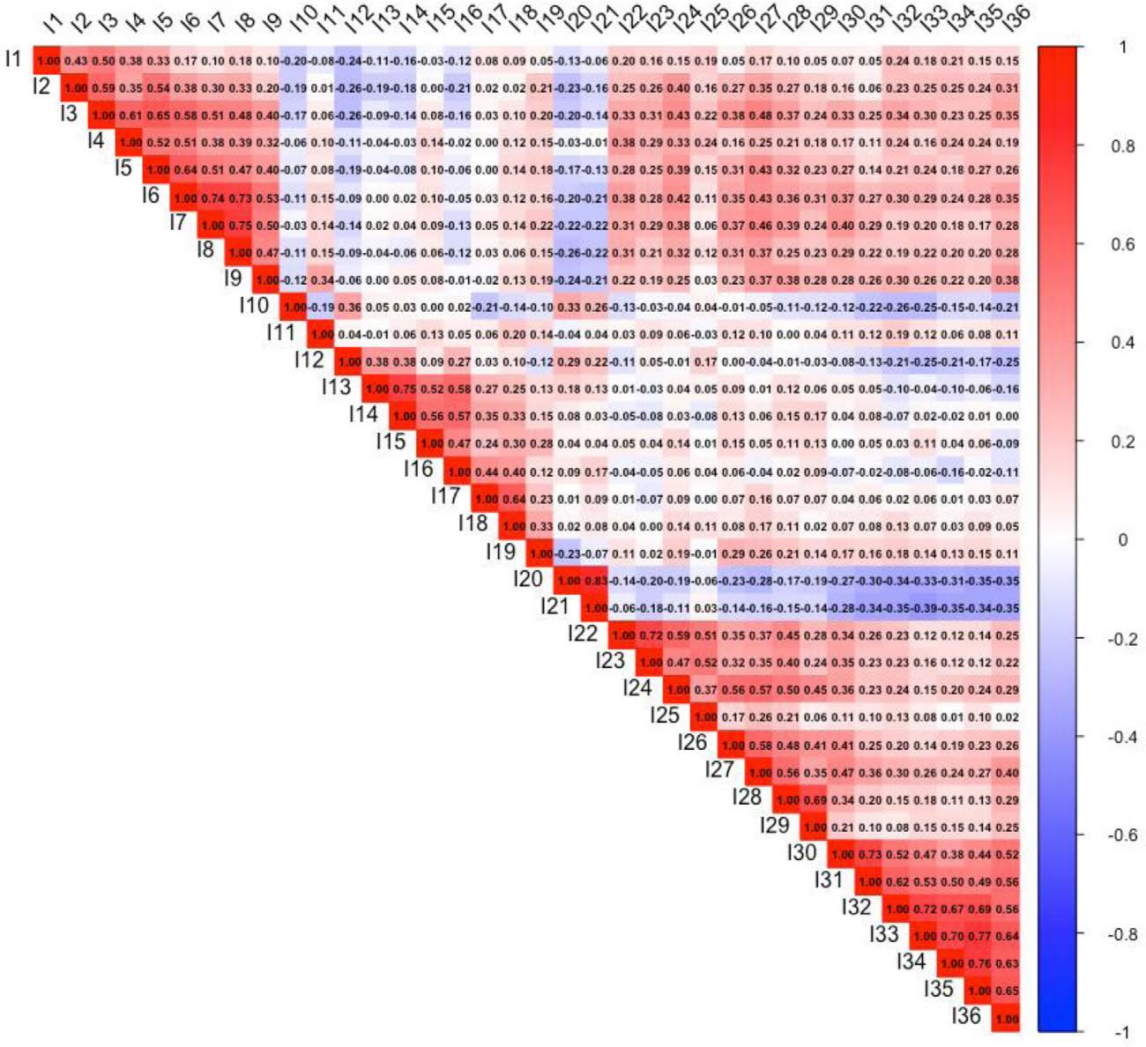
Correlation heatmap of survey items.

The correlation heatmap presented in Figure 1 illustrates the interrelationships among survey items (I1–I36). The Pearson correlation coefficients indicate the strength and direction of associations, where values closer to + 1 represent strong positive correlations, values closer to − 1 indicate strong negative correlations, and values near 0 suggest little to no relationship. The color gradient provides a visual representation, with red shades denoting positive correlations and blue shades indicating negative correlations. The strong clustering of correlations among specific question groups suggests the potential for underlying latent factors. These findings can provide valuable insights for further confirmatory factor analysis to validate the scale structure. Moreover, items with consistently weak correlations across the matrix may be candidates for revision or removal to enhance the robustness of psychometric properties of the scale.

After conducting a confirmatory factor analysis on the full model, items I10, I11, and I12 were found to have low factor loadings (−0.244, 0.225, and −0.281, respectively) and statistically nonsignificant p-values (*P* = .331, 0.494, and 0.517, respectively), indicating that they did not adequately represent the colleague support dimension. Consequently, these items were removed, and the model was reevaluated. The modified model exhibited improved fit indices, including higher comparative root indices (0.880 vs 0.845), higher (0.87 vs 0.86), higher average inter-item correlation (0.18 vs 0.14) and lower root mean square error of approximation indices (0.070 vs 0.074), suggesting a better overall model fit.

**Figure 2.**
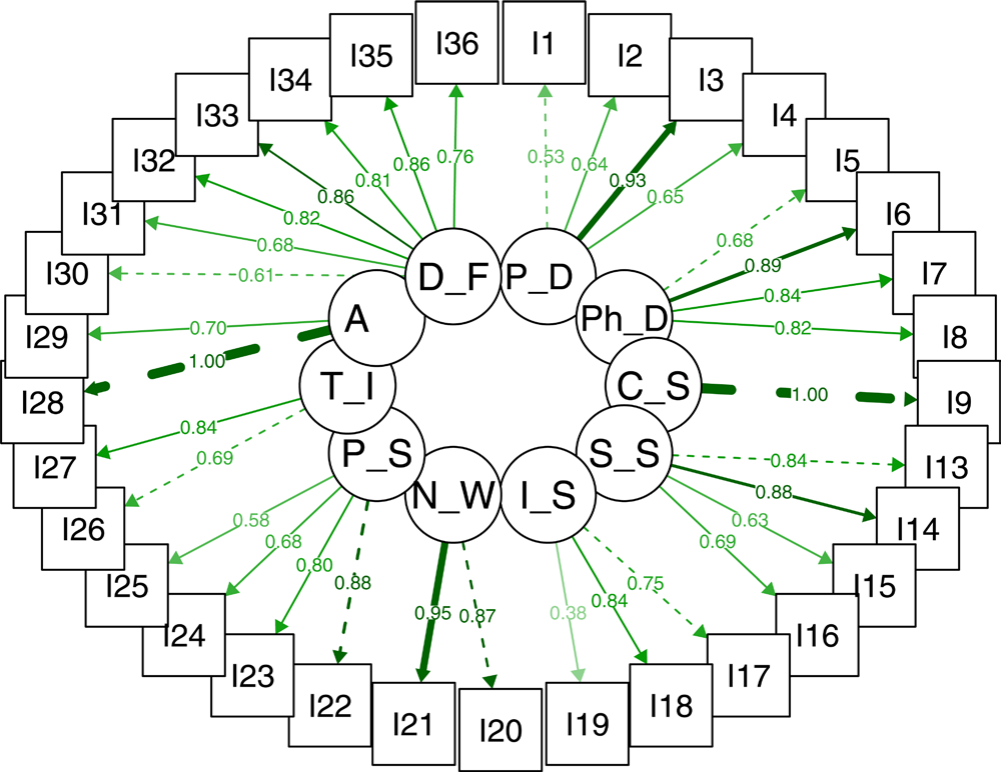
Structural equation model of T-SVEST data. A = absenteeism, CS = colleague support, DF = desired forms of support, LS = institutional support, NW = nonwork-related support, PD = psychological distress, PhD = physical distress, PS = professional self-efficacy, SS = supervisor support, T-SVEST = Turkish adaptation of second-victim experience and support tool, TI = turnover intentions.

This structural equation model in Figure 2 visualizes the relationships between dimensions (latent variable) and items. The rectangles represent survey items while the ellipses denote the dimensions that are inferred from these items. The arrows indicate the relationships between the dimensions and items. The numbers on the arrows represent factor loadings, which indicate the strength of the relationship between each latent variable and its observed items. Higher loadings suggest stronger associations. For example, if an item related to Psychological Distress (P_D) has a high loading, it strongly contributes to the overall construct.

The Overall SVEST Score, calculated based on 26 items, had a mean of 75.71 and a standard deviation of 15.12. The desired forms of support dimension was excluded from its calculation to maintain conceptual clarity, as it represents future expectations rather than current experiences.

**Figure 3.**
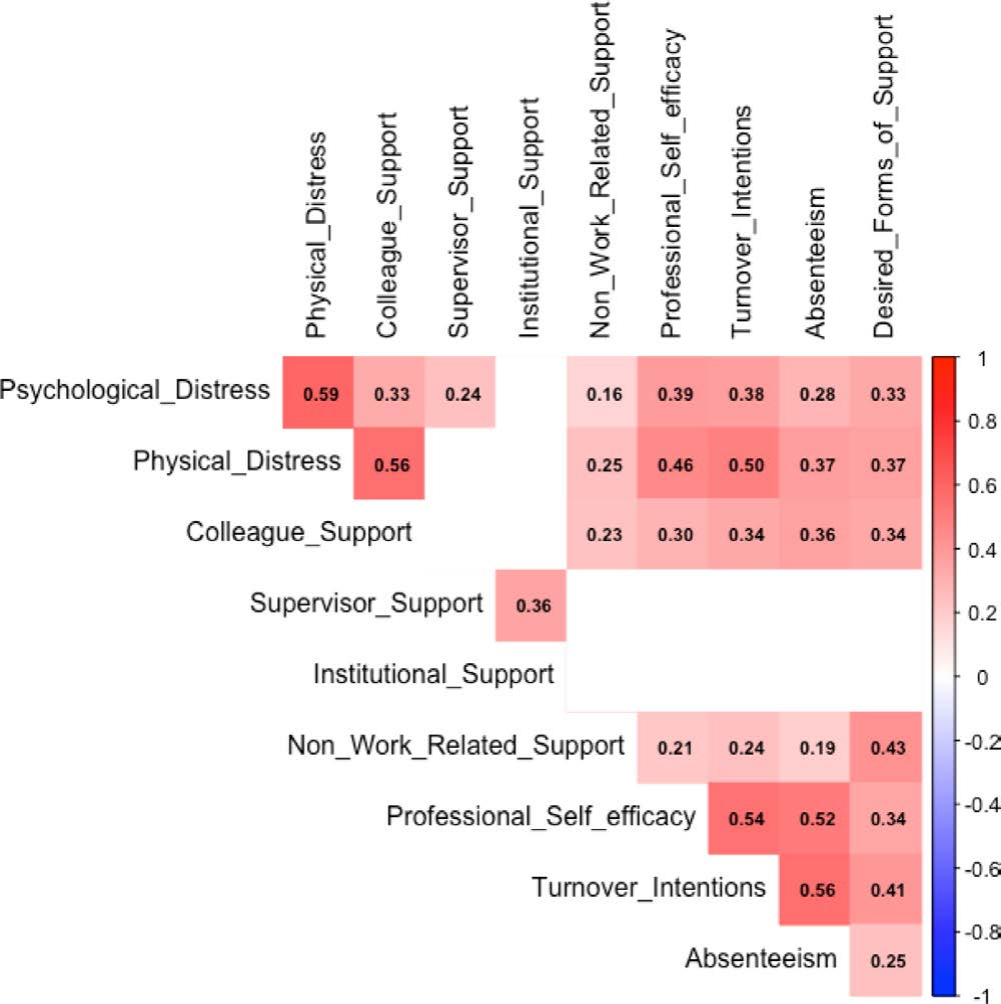
Correlation plot of the dimensions.

The correlation plot shown in Figure 3 visually represents the statistically significant relationships (*P* < .05) between key study variables, while nonsignificant associations are omitted for clarity. Among the notable findings, psychological distress is significantly correlated with physical distress (*R* = 0.59), colleague support (*R* = 0.33), and professional self-efficacy (*R* = 0.39), indicating that individuals experiencing higher distress also report increased physical symptoms and self-perceived competence. Similarly, physical distress is positively associated with professional self-efficacy (*R* = 0.46), turnover intentions (*R* = 0.50), and absenteeism (*R* = 0.37), suggesting that individuals with greater physical distress may experience more job-related strain and a higher likelihood of leaving their position. supervisor support shows a significant correlation with turnover intentions (*R* = 0.36), emphasizing its potential role in employee retention. Additionally, professional self-efficacy is strongly correlated with Turnover Intentions (*R* = 0.54) and absenteeism (*R* = 0.52), reinforcing its role in workplace behavior. Nonwork-related support is positively associated with desired forms of support (*R* = 0.43), suggesting that individuals who receive external support also express a greater need for workplace support. The blank spaces in the matrix indicate nonsignificant correlations (*P* > .05), signifying relationships that were not statistically meaningful.

In addition to examining the correlations between dimensions; *t*-tests, 1-way ANOVA, and Tukey HSD tests were conducted to explore the effects of demographic and socioeconomic variables on these dimensions.

A statistically significant association was found between colleague support and work experience (*P* = .015). post hoc Tukey comparisons indicated that participants with 21 to 30 years of experience reported significantly higher levels of colleague support than those with 0 to 10 (*P* = .029) and 11 to 20 years (*P* = .038) years of experience. Similarly, educational status had a significant effect on institutional support (*P* = .01). post hoc analysis showed that individuals with a bachelor’s degree reported significantly lower institutional support compared to those with an associate degree (*P* = .029). Regarding physical distress, a significant difference was observed between married and single participants (*P* = .042), with married individuals reporting lower levels of distress compared to their single counterparts. Finally, age was positively correlated with colleague support (*R* = 0.378), suggesting that older participants reported receiving greater support from colleagues. On the other hand, apart from these relationships, no significant effects of demographic and socioeconomic variables were found on the remaining dimensions. Lastly, the analysis of overall experience did not reveal any significant effects for age (*P* = .870), marital status (*P* = .137), educational status (*P* = .166), occupation (*P* = .988), work experience (*P* = .650), or organization (*P* = .598).

## 4. Discussion

This study explored the prevalence and dimensions of second-victim experiences among anesthesia and intensive care professionals in Türkiye, utilizing the T-SVEST. The findings provide critical insights into the psychosocial impact of adverse events on healthcare workers in high-risk clinical settings and contribute to the growing international body of evidence on the second-victim phenomenon as well as underscoring the urgent need for systemic institutional support.

### 4.1. Psychometric validity and reliability

The T-SVEST demonstrated good psychometric properties, with a high overall internal consistency (Cronbach α = 0.8623), supporting its reliability in capturing second-victim dimensions. However, item-level analysis revealed low MSA values and weak loadings for certain items (e.g., I10–I12), particularly in the Colleague Support dimension. These inconsistencies are not unique to our sample; similar issues have been reported in other adaptation studies where Persian,^[11]^ Malaysian,^[7]^ and Danish^[8]^ versions of SVEST were used. The removal of underperforming items improved the model fit indices, which aligns with recommendations from recent literature advocating for flexible item refinement based on local context.^[9,12]^

### 4.2. Prevalence and symptom dimensions

Consistent with the literature,^[1,13,14]^ our findings confirm the frequent occurrence of psychological and physical distress among healthcare professionals following adverse events, manifesting in symptoms akin to post-traumatic stress disorder-such as guilt, anxiety, and grief-which can undermine self-efficacy and confidence in their professional abilities. Psychological distress in our study showed a strong correlation with physical distress and professional self-efficacy, suggesting that emotional burden and occupational functioning are closely intertwined. These factors are of particular concern in anesthesia and ICU settings, where the gravity of decisions can lead to severe repercussions not only for patients but also for the medical staff involved.^[15,16]^ Given the strategic importance of retaining experienced anesthesia and intensive care professionals (especially in resource-constrained settings) developing targeted interventions to support affected clinicians is both a humanistic and operational imperative. Indeed, our findings draw attention to the need for more proactive and visible support systems from an institutional perspective. The relatively high scores on supervisor and institutional support dimensions indicate perceived inadequacy in organizational response to adverse events.

Likewise, physical distress significantly correlated with turnover intentions and absenteeism findings that echo the data released in studies from Spain,^[17]^ Austria,^[18]^ and Argentina.^[5]^ These correlations highlight the potential organizational consequences of unaddressed second-victim experiences, including workforce attrition, presenteeism, and diminished team morale.^[12,19]^

### 4.3. Role of support mechanisms

The perceived inadequacy of institutional, supervisor, and colleague support in our sample is consistent with findings from studies in pediatric and adult care settings.^[10,15,18]^ Participants with more years of experience and those of older age reported higher levels of colleague support – a phenomenon potentially linked to stronger peer networks and adaptive coping skills developed over time.^[20,21]^ However, less experienced staff – those arguably more vulnerable to psychological trauma – reported significantly lower support levels, reaffirming the importance of mentoring and structured debriefing processes.^[22,23]^

Our results also showed that married professionals reported significantly lower levels of physical distress, possibly reflecting the buffering role of social support from family-a finding supported by studies on nonwork-related resilience.^[24,25]^ This finding could also signal the protective role of personal relationships in coping with workplace stressors.^[26]^ These personal resources may mitigate the emotional toll of second-victim experiences, but cannot replace systemic organizational interventions.

The high demand for peer and professional counseling services among our participants is consistent with international preferences for peer-driven models, such as the RISE (Resilience in Stressful Events) support program established by Johns Hopkins University, and HELP (The Healing the Emotional Lives of Peers) program launched by Mayo Clinic.^[15]^ These structured, multi-tiered frameworks have demonstrated positive effects on both psychological outcomes and perceptions of institutional culture.^[12,17]^

### 4.4. Sociodemographic insights

While prior studies have noted a greater psychological toll on younger and less experienced clinicians,^[4,11]^ our findings suggest that distress is widely distributed across different age groups and professional backgrounds. Interestingly, a significant proportion of our participants were experienced specialists, nurses, or academic physicians with more than 10 years of experience in practice, highlighting that second-victim experiences are not confined to early-career professionals. Participants with greater work experience reported higher levels of colleague support which could suggest that seasoned professionals may have developed strategies for navigating the emotional landscape following adverse events, often relying on established relationships within their workplace. This leads to the hypothesis that mentorship programs that foster supportive interactions could be beneficial for less experienced staff who may feel overwhelmed or unsupported in the wake of adverse incidents.^[23]^

Although most demographic factors such as gender, occupation, or workplace type did not significantly influence the overall SVEST scores, differences in perceived support across educational and experience levels suggest institutional blind spots in support allocation. The nuances of these findings underscore the importance of tailored support interventions that consider individual backgrounds and social contexts.^[20,27]^ While some studies suggest that certain personality traits (e.g., neuroticism) increase susceptibility to second-victim stress,^[18,28]^ such factors were not examined in our dataset. Future research in Türkiye should consider incorporating validated personality and resilience scales to better understand intrapersonal moderators of second-victim responses.

### 4.5. Strategic implications

From an organizational standpoint, the findings reinforce the importance of embedding second-victim support into broader patient safety and quality improvement agendas. Institutions should adopt a dual approach such as reactive support after adverse events and proactive education and training to build psychological readiness.^[12,19]^ Evidence-based interventions such as training plans,^[12]^ after-action reviews,^[29]^ and staff-led debriefing should become routine components of safety culture frameworks. It is also necessary to consider organizations’ cultural attitudes towards errors and the available mechanisms for support and recovery. A culture that stigmatizes errors rather than promoting learning and support significantly affects how second-victims experience and process these events.^[17,19]^ The literature suggests that in environments characterized by psychological safety, healthcare professionals are more likely to seek help, thereby reducing the risk of sustained emotional distress and fostering resilience in the face of adversity.^[18,25]^ This approach necessitates an urgent call to action for healthcare organizations to integrate peer support programs and establish comprehensive training that prepares staff to manage the aftermath of adverse events effectively.^[29,30]^

The evidence presented in this study adds to the growing body of literature advocating for a systemic approach to the second-victim phenomenon, combining qualitative and quantitative insights to craft more effective intervention strategies. The need for continued investigation into the long-term impacts of second-victim experiences and outcomes remains paramount. Given the documented prevalence of second-victim syndrome, further longitudinal studies could yield insights into the efficacy of peer support initiatives over time and their impact on both personal and professional development.^[21,31]^

Additionally, aligning these efforts with international definitions and best practices, as proposed by the European Cooperation in Science and Technology (COST) Action 19113,^[19]^ could enhance transparency, trust, and inter-professional solidarity across the healthcare ecosystem.

## 5. Conclusion

This study contributes valuable insights into the prevalence, structure, and correlates of second-victim experiences among anesthesia and intensive care professionals in Türkiye. By employing the T-SVEST, we have demonstrated that psychological and physical distress remain highly prevalent in this population, alongside a substantial desire for structured support mechanisms, particularly peer support. The study also affirmed the T-SVEST as a reliable and valid instrument for measuring second-victim phenomena in this specific cultural and clinical context.

Our findings suggest that current institutional and supervisory support systems may be insufficient, further reinforcing the need for healthcare organizations to proactively implement evidence-based peer support frameworks and training programs to respond to the needs of clinicians impacted by adverse events. Recognizing and addressing the second-victim phenomenon is not only a matter of individual psychological well-being but also of patient safety, workforce sustainability, and organizational resilience. Future research should prioritize the longitudinal tracking of second victim outcomes and the implementation of intervention models tailored to the specific needs of healthcare professionals working in anesthesiology and intensive care settings. With increasing awareness and appropriate systemic support, it is possible to mitigate the long-term consequences of second victim distress and cultivate a culture of empathy, learning, and safety in healthcare institutions.

## Author contributions

**Conceptualization:** Pinar Ayvat, Ozlem Oner, Gulsah Sehitoglu Alpagut.

**Data curation:** Pinar Ayvat, Ali Galip Ayvat, Ozlem Oner, Gulsah Sehitoglu Alpagut.

**Formal analysis:** Pinar Ayvat, Ali Galip Ayvat, Gunal Bilek, Ozlem Oner.

**Funding acquisition:** Omer Faruk Sonmez.

**Investigation:** Pinar Ayvat, Ozlem Oner, Gulsah Sehitoglu Alpagut.

**Methodology:** Pinar Ayvat, Ali Galip Ayvat, Gunal Bilek, Ozlem Oner, Gulsah Sehitoglu Alpagut.

**Project administration:** Ali Galip Ayvat, Ozlem Oner.

**Resources:** Gulsah Sehitoglu Alpagut, Omer Faruk Sonmez.

**Software:** Gunal Bilek, Omer Faruk Sonmez.

**Supervision:** Pinar Ayvat, Ali Galip Ayvat, Gulsah Sehitoglu Alpagut.

**Validation:** Pinar Ayvat, Ali Galip Ayvat, Gunal Bilek, Ozlem Oner, Gulsah Sehitoglu Alpagut.

**Visualization:** Ali Galip Ayvat, Gunal Bilek, Ozlem Oner, Omer Faruk Sonmez.

**Writing – original draft:** Ali Galip Ayvat, Gunal Bilek, Ozlem Oner.

**Writing – review & editing:** Pinar Ayvat, Ali Galip Ayvat, Gunal Bilek, Ozlem Oner, Gulsah Sehitoglu Alpagut, Omer Faruk Sonmez.
